# Seroprevalence and genetic characterization of *Toxoplasma gondii* in three species of pet birds in China

**DOI:** 10.1186/1756-3305-7-152

**Published:** 2014-04-01

**Authors:** Wei Cong, Qing-Feng Meng, Hui-Qun Song, Dong-Hui Zhou, Si-Yang Huang, Ai-Dong Qian, Chunlei Su, Xing-Quan Zhu

**Affiliations:** 1 State Key Laboratory of Veterinary Etiological Biology, Key Laboratory of Veterinary Parasitology of Gansu Province, Lanzhou Veterinary Research Institute, Chinese Academy of Agricultural Sciences, Lanzhou, Gansu Province 730046, PR China; 2 College of Animal Science and Technology, Jilin Agricultural University, Changchun, Jilin Province 130118, PR China; 3 Jilin Entry-Exit Inspection and Quarantine Bureau, Changchun, Jilin Province 130118, PR China; 4 Department of Microbiology, the University of Tennessee, Knoxville, TN 37996, USA

**Keywords:** *Toxoplasma gondii*, Toxoplasmosis, Pet birds, Seroprevalence, Genetic characterization

## Abstract

**Background:**

Toxoplasmosis, caused by the protozoan parasite *Toxoplasma gondii,* is one of the most common zoonosis worldwide, affecting a wide range of warm-blooded mammals and birds worldwide. However, no information on *T. gondii* infection in pet birds in China is available. Therefore, this study was performed to determine the prevalence of *T. gondii* infection in pet birds in Gansu province, China.

**Methods:**

A total of 687 blood samples were collected from pet birds (*Carduelis spinus, Alauda gulgula, Cocothraustes migratorlus*) in three representative administrative regions in Gansu province, northwest China between August 2011 and September 2012 *T. gondii* antibodies were determined using the modified agglutination test (MAT). Genomic DNA was extracted from the brain tissues of seropositive pet birds and *T. gondii* B1 gene was amplified using a semi-nested PCR.DNA samples giving positive B1 amplification were then genetically characterized using multi-locus PCR-RFLP.

**Results:**

The overall *T. gondii* seroprevalence was 11.21% (77/687). *C. spinus* had the highest *T. gondii* seroprevalence (11.65%), followed by *A. arvensis* (11.39%) and *C. migratorlus* (5.26%), these differences were not statistically significant (*P* > 0.05). Of 77 DNA samples, 8 were positive for the *T. gondii* B1 gene, four showed complete genotyping results. Only one genotype (the Type II variant: ToxoDB genotype #3) was identified.

**Conclusions:**

The results of the present survey indicated the presence of *T. gondii* infection in pet birds in Gansu province, China. These data provide base-line information for the execution of control strategies against *T. gondii* infection in pet birds. To our knowledge, this is the first report documenting the occurrence of *T. gondii* prevalence and genotype in pet birds in China.

## Background

Toxoplasmosis is one of the most common zoonosis worldwide caused by the protozoan parasite *Toxoplasma gondii*. It is estimated that *T. gondii* infects up to one-third of the human population in the world [1]. Humans are infected by ingesting oocysts shed by cats or consuming under-cooked meat with parasite tissue cysts [2]. It can cause severe disease in the fetus during congenital infection, and can be fatal to immunocomprimised patients such as those with AIDS or organ transplant [3,4]. *T. gondii* infects a wide range of animals, including mammals and birds. In spite of the high prevalence of toxoplasmosis reported for several species of wild birds around the world [5], there is yet no information in pet birds.

Pet birds are very important to public health because they may act as natural reservoirs for many pathogens, such as Newcastle disease virus, Avian influenza virus, *Chlamydia psittaci* and others pathogens [6-9], and they have also been proved to be important infection sources for a variety of etiologic agents. In China, pet birds have been raised and kept over a long period of history for companionship and entertainment, and they maintain close contact with humans, especially in urban areas. Urban areas provide a particular and complicated environment where closely interacting biological and social constraints may either directly or indirectly impact on the presence of *T. gondii* in pet birds, particularly, pet birds are frequently taken to the park by their owners, can easily come into contact with the stray cats and stray dogs. Usually, *T. gondii* infection in pet birds is not harmful for humans. However, dead pet birds both from pet shops and from households are often thrown away without any bio-safety considerations. The dead birds may be eaten by cats and, if the birds contained *T. gondii,* the cats may become infected with the parasite and subsequently shed oocysts. Humans can become infected postnatally via ingesting *T. gondii* tissue cysts from water, vegetables, fruits and other food contaminated with *T. gondii* oocysts. In addition, pet birds share nearly the same environment with people in urban areas, such as water, food and others, so the investigation of *T. gondii* infection in pet birds might be suitable to estimate the risk of *T. gondii* infection in urban residents. In China, Eurasian Siskin (*Carduelis spinus*), Oriental Skylark (*Alauda gulgula*) and Black-tailed Grosbeak (*Cocothraustes migratorlus*) are very popular as pet birds. Here we conducted a study on the seroprevalence and genotyping of *T. gondii* in these pet birds in Gansu province, China.

## Methods

### Ethics statement

The present study was approved by the Animal Ethics Committee of Lanzhou Veterinary Research Institute, Chinese Academy of Agricultural Sciences (Permit code LVRIAEC2011-009). Birds from which serum samples were collected were handled in accordance with the Animal Ethics Procedures and Guidelines of the People’s Republic of China.

### The site

The present investigation was carried out in Gansu province, China. Gansu has an area of 454,000 square kilometres, and the vast majority of its land is more than 1,000 meters above sea level. It lies between the Tibetan Plateau and the Loess Plateau, the province contains the geographical center of China, marked by the Center of the Country Monument at 35°50′40.9′N 103°27′7.5″E. Gansu generally has a semi-arid to arid, continental climate, with warm to hot summers and cold to very cold winters. Most of the precipitation is delivered in the summer months.

### Collection and preparation of serum samples

A total of 687 blood samples were collected between August 2011 and September 2012 from pet birds of three species in three representative administrative regions in Gansu province, China (Table 1). All bird samples from pet markets were collected randomly, and one blood sample was collected from each bird. Blood samples were then centrifuged at 3,000 *g* for 10 min, and serum was collected, frozen, and stored at -20°C until use.

**Table 1:** **Prevalence of *Toxoplasma gondii *** **infection in three species of pet birds ( *Carduelis spinus *, *Alauda gulgula *** **and *Cocothraustes migratorlus*) in China**

**Host**	**Locations**	**No. of sera with MAT titers of**						**No. examined**	**No. positive**	**Prevalence (%)**
		**1:5**	**1:10**	**1:20**	**1:40**	**1:80**	**1:160**			**(95% CI)**
*C. spinus*	Lanzhou	5	4	6	2	2	0	120	19	15.83 (9.30-22.37)
	Tianshui	4	2	2	2	0	0	120	10	8.33 (3.89-13.28)
	Longnan	1	8	2	0	1	0	120	12	10.00 (4.63-15.37)
*A. gulgula*	Lanzhou	5	3	3	3	2	1	135	17	12.59 (7.00-18.19)
	Tianshui	2	2	3	4	0	0	73	11	15.07 (6.86-23.28)
	Longnan	1	1	3	1	0	0	81	6	7.41 (1.70-13.11)
*C. migratorlus*	Tianshui	1	1	0	0	0	0	38	2	5.26 (0.00-12.36)
Total		19	21	19	12	5	1	687	77	11.21 (8.85-13.57)

### Serological examination

The pet bird serum samples were tested for *T. gondii* antibodies by the modified agglutination test (MAT) as described previously [10,11]. Briefly, sera were added to the "U" bottom of 96-well microtiter plates, and diluted 2-fold starting from 1:5 to 1:160. Bird sera with MAT titers of 1:5 or higher were considered positive for *T. gondii* antibodies based on a previous study [12], those sera with doubtful reactions were re-tested, and positive and negative controls were included in each test.

### DNA extraction and genetic characterization of *T. gondii* isolates

The brain tissues of seropositive pet birds were used for DNA extraction. Genomic DNA was extracted from these tissues using TIANamp Genomic DNA kit (TianGen™, Beijing, China) according to manufacturer’s recommendations. In brief, 30 mg of the tissues were treated with sodium dodecyl sulphate/proteinase K at 56°C for 5 h digestion in a thermostat water bath. DNA samples were prepared after purification by silica gel column chromatography and eluted into 50 μl elution buffer. Then, a semi-nested PCR targeting the *T. gondii* B1 gene was performed to detect possible infection with *T. gondii*[13]. DNA samples giving positive B1 amplification were then used for genetic characterization. Genotyping was conducted using 10 genetic markers for PCR-RFLP (i.e., SAG1, SAG2, alter.SAG2, SAG3, BTUB, GRA6, c22-8, L358, PK1, and Apico), as previously described [14-18]. Six reference *T. gondii* strains were included as the positive controls including GT1, PTG, CTG, MAS, TgCgCa1 and TgCatBr5. The PCR reaction (25 μl) composed of 1× PCR buffer, 0.2 mM of each primer, 200 μM dNTPs, 2 mM MgCl_2_, 0.2 U of HotStart *Taq* DNA polymerase (TAKARA, Japan). The PCR amplification was performed using a thermal cycler (PTC 200, Bio-RAD). All samples were incubated at 95°C for 5 min to activate the DNA polymerase, then 30 cycles of PCR at 95°C for 30 s, 55°C for 60 s and 72°C for 90 s. Multiplex PCR-amplified products were diluted 1:1 in sterile, double-distilled water, and then used for nested PCR amplifications with internal primers for each marker, separately [19,20]. A similar program was used for the nested PCR. The nested PCR amplifications were performed with the annealing temperature at 60°C for 60 s for all the markers except Apico, which was amplified at 55°C. The nested PCR products were digested with restriction enzymes for 1 h, and the temperature for each enzyme was used according to the instructions for each enzyme. The restriction fragments were resolved in 2.5%-3% agarose gel, stained by the GoldenView™, and photographed using a gel documentation system (UVP GelDoc-ItTM Imaging System, Cambridge, U.K.).

### Statistical analysis

Differences in the seroprevalence of *T. gondii-*infected pet birds among different variables including location, age, gender and species were analyzed using a Chi-square test by SAS (Statistical Analysis System, Version 8.0). Results were considered statistically significant when *P* < 0.05. These variables were also evaluated in the binary Logit model as independent variables by forward stepwise regression analysis to test the seroprevalence (response variable) in the multivariable regression analysis. The best model was judged by the Fisher’s scoring algorithm. The effects could be included in the model when *P* < 0.05.

## Results

The overall seroprevalence of *T. gondii* in the examined pet birds was 11.21% (77/687, 95% confidence interval [CI] 8.85-13.57). Of these, *A. gulgula* had the highest *T. gondii* seroprevalence (11.77%, 95% CI 8.05-15.48), followed by *C. spinus* (11.39%, 95% CI 8.11-14.67) and *C. migratorlus* (5.26%, 95% CI 0.00-12.36).

No statistically significant difference was found in the seroprevalence of *T. gondii* between male pet birds (10.84%, 95% CI 7.85-13.84) and female pet birds (11.77%, 95% CI 7.94-15.5, *P* = 0.708). The prevalence in adult pet birds (13.20%, 95% CI 10.28-16.13) was significantly higher than that in juvenile pet birds (5.23%, 95% CI 1.91-8.56, OR = 2.76, 95% CI 1.34-5.65, *P* = 0.004). Statistical analysis of the originating regions showed that pet birds from Lanzhou had a higher *T. gondii* seropositivity (14.12%, 95% CI 9.84-18.39) than the birds from Tianshui (9.96%, 95% CI 6.10-13.82) and Longnan (8.96%, 95% CI 5.01-12.90), but the difference was not statistically significant (*P* = 0.169) (Table 2). In the multivariable regression analysis, no risk factors could be included in the final model because all the *P* values were above 0.05.

**Table 2:** **Analysis of the variables associated with *Toxoplasma gondii *** **seroprevalence in three species of pet birds ( *Carduelis spinus *, *Alauda gulgula *** **and *Cocothraustes migratorlus*) in China**

**Variable**	**Category**	**No. tested**	**No. positive**	**% (95% CI)**	***P *- value**	**OR (95%CI)**
Region	Longnan	201	18	8.96 (5.01-12.90)	0.169	Reference
	Tianshui	231	23	9.96 (6.10-13.82)		1.12 (0.59-2.15)
	Lanzhou	255	36	14.12 (9.84-18.39)		1.67 (0.92-3.04)
Gender	Male	415	45	10.84 (7.85-13.84)	0.708	Reference
	Female	272	32	11.77 (7.94-15.59)		1.10 (0.68-1.77)
Species	*Cocothraustes migratorlus*	38	2	5.26 (0.00-12.36)	0.484	Reference
	*Alauda gulgula*	289	34	11.77 (8.05-15.48)		2.40 (0.55-10.42)
	*Carduelis spinus*	360	41	11.39 (8.11-14.67)		2.31 (0.54-9.97)
Age	Juvenile	172	9	5.23 (1.91-8.56)	0.004	Reference
	Adult	515	68	13.20 (10.28-16.13)		2.76 (1.34-5.65)
Total		687	77	11.21 (8.85-13.57)		

95% CI: 95% confidence interval.

OR: Odds ratios.

Of 77 DNA samples, 8 were positive for the *T. gondii* B1 gene, including 5 from the *C. spinus* and 3 from the *A. gulgula*. Four DNA samples showed complete genotyping results, 3 from Lanzhou (2 from *C. spinus* and 1 from *A. gulgula*) and 1 from Tianshui (from *A. gulgula*) (Table 3). Another 4 positive samples could not be completely genotyped due to low DNA concentration. Interestingly, only one genotype was identified from the 4 positive samples, which were typed at 10 genetic markers, including 9 nuclear loci, i.e., SAG1, 5′-and 3′-SAG2, alternative SAG2, SAG3, GRA6, BTUB, L358, PK1, c22-8 and one an apicoplast locus Apico. These Type II variant strains had type II alleles at all markers except a type I allele at the Apico locus and are considered the Type II variant (ToxoDB genotype #3).

**Table 3:** **Multilocus genotyping of *Toxoplasma gondii *** **isolates in pet birds from China by PCR-RFLP analysis**

**Isolate ID**	**Host**	**Location**	**SAG1**	**5′ + 3′ SAG2**	**Alt. SAG2**	**SAG3**	**BTUB**	**GRA6**	**c22-8**	**L358**	**PK1**	**Apico**	**Genotype**
GT1	Goat	United States	I	I	I	I	I	I	I	I	I	I	Reference, Type I, ToxoDB #10
PTG	Sheep	United States	II/III	II	II	II	II	II	II	II	II	II	Reference, Type II, ToxoDB #1
CTG	Cat	United States	II/III	III	III	III	III	III	III	III	III	III	Reference, Type III, ToxoDB #2
MAS	Human	France	u-1^*^	I	II	III	III	III	u-1^*^	I	III	I	Reference, ToxoDB #17
TgCgCa1	Cougar	Canada	I	II	II	III	II	II	II	I	u-2^*^	I	Reference, ToxoDB #66
TgCatBr5	Cat	Brazil	I	III	III	III	III	III	I	I	u-1^*^	I	Reference, ToxoDB #19
LZES125	Eurasian Siskin	Lanzhou, China	II	II	II	II	II	II	II	II	II	I	Type II variant, ToxoDB #3
LZES260	Eurasian Siskin	Lanzhou, China	II	II	II	II	II	II	II	II	II	I	Type II variant, ToxoDB #3
LZOS124	Oriental Skylark	Lanzhou, China	II	II	II	II	II	II	II	II	II	I	Type II variant, ToxoDB #3
TSOS188	Oriental Skylark	Tianshui, China	II	II	II	II	II	II	II	II	II	I	Type II variant, ToxoDB #3

* u-1 and u-2 represent unique RFLP genotypes, respectively.

## Discussion

In this investigation, the overall seroprevalence of *T. gondii* in the examined pet birds was 11.21% (77/687), which was significantly different from that in wild birds worldwide [5]. The differences in seroprevalences of *T. gondii* in pet birds are probably due to differences in geographical conditions, feeding and living styles, number of cats and rodents, as well as differences in animal husbandry practices and animal welfares.

The *T. gondii* seroprevalence in adult pet birds (13.20%, 68/515, 95% CI 10.28-16.13) was significantly higher than that in juvenile pet birds (5.23%, 9/172, 95% CI 1.91-8.56, OR = 2.76, 95% CI 1.34-5.65, *P* = 0.004), this result is probably due to the fact that the adult pet birds had more chance to contact with *T. gondii* compared to juvenile pet birds and thus increased the risk of infection, demonstrating that the adult pet birds may significantly increase the probability of *T. gondii* infection.

This result provides evidence that Type II variant genotype prevails in bird populations in mainland China, as recently reported [21]. It is interesting that most *T. gondii* isolates reported in previous studies represent the predominant ToxoDB #9 in China [21-23]. It seems that this dominant genotype is mostly in the east and southeast regions in China, suggesting population differences among different geographical regions. More studies are needed to investigate this potential difference and the mechanisms that leads to the population structure.

Previous studies [1,10-12,15] have demonstrated that MAT is a sensitive method for measuring antibodies to *T. gondii* in birds and is considered as an effective serologic method as a guide on isolating *T. gondii* from tissues of birds. In the present study, the antibody titres were diverse; the most frequent level was 1:10 (27.27%), followed by 1:5 (24.68%), 1:20 (24.68%), 1:40 (15.58%), 1:80 (6.49%) and 1:60 (1.30%). PCR with specific primers is regarded as a sensitive and cost-effective assay for detecting *T. gondii* DNA from biological samples directly [19]. In this study, a semi-nested PCR targeting the *T. gondii* B1 gene was performed to detect possible infection with *T. gondii.* Of 77 DNA samples, 8 were positive for the *T. gondii* B1 gene, including 5 from the *C. spinus* (1 with MAT titer at 1:10, 2 at 1:40, 2 at 1:80) and 3 from the *A. gulgula* (2 at 1:40, 1 at 1:80). Since no *T. gondii* strain was isolated, the PCR result itself is suggestive, but not the ultimate evidence of infection. Therefore, the results of the present study provided preliminary data for future studies aimed at isolating live parasites in pet birds and confirm the findings.

Humans can acquire *T. gondii* infection from domestic, wild or companion animals [1-4]. Although pets bring a lot of fun and enrich family life, they also pose risks of infectious disease for humans. Due to the current irregular management of pet markets, lack of quarantine and preventive measures, pet diseases continue to spread, and there have been reports of human infection [24-28]. Generally, direct transmission of *T. gondii* from pet birds to humans is unlikely to occur, since the parasites are confined in internal organs and muscles [15,18]. In addition, these pet birds are not usually bred to produce meat for food, therefore they are not likely to transmit *T. gondii* to humans [29]. Surprisingly, during the investigation, we found that the pet shop workers discard dead pet birds as trash without any biosafety measures. In addition, many cats (pet cats and stray cats) are often seen in the pet shops, so they could easily ingest the pet birds due to staff negligence. Once cats and other felids ingest the infected pet birds (alive or dead), tens of millions of oocysts can be shed into the environment in several days, which can potentially infect a variety of animals in large numbers [30], which is a huge safety risk for urban residents.

Epidemiological knowledge regarding the prevalence and risks associated with *T. gondii* infection in pet birds is unavailable. In China, most pet birds are bred in a semi-free range system, and the birds have opportunities to contact food or water contaminated with *T. gondii.* The results of the present survey revealed the presence of *T. gondii* infection in pet birds in China, which indicated that pet birds may be new reservoirs of *T. gondii*. Pet birds are among the common companion animals of humans. These results indicate existence of this parasite in their environment, which may also pose a risk for human infection. Moreover, investigating the health status of pet birds, facilities, pet shop workers and owners should be an important starting point to evaluate human health risks for zoonotic diseases, and to establish disease control and prevention measures. It is imperative that effective measures for prevention and better control of *T. gondii* is needed for pet animals. To our knowledge, this is the first report documenting the occurrence of *T. gondii* prevalence in pet birds in China.

## Conclusions

The present study revealed the prevalence of *T. gondii* prevalence in pet birds in China for the first time, and identified the *T. gondii* Type II variant strain (ToxoDB genotype #3). These data may provide base-line information for the control *T. gondii* infection in pet birds in China.

## Competing interests

The authors declare that they have no competing interests.

## Authors’ contributions

XQZ and CS conceived and designed the study, and critically revised the manuscript. WC, QFM, HQS and DHZ performed the experiments, analyzed the data and drafted the manuscript. SYH and ADQ helped in study design, study implementation and manuscript revision. All authors read and approved the final manuscript.
